# Editorial: The Role of Vitamin D in Gut Health and Disease in Children

**DOI:** 10.3389/fpubh.2022.912773

**Published:** 2022-05-11

**Authors:** Andrew S. Day, Abdulbari Bener, Ihab Tewfik, Pietro Vajro, Susu M. Zughaier

**Affiliations:** ^1^Department of Paediatrics, University of Otago Christchurch, Christchurch, New Zealand; ^2^Departments of Biostatistics & Medical Informatics and Public Health, Istanbul Medipol University, Istanbul, Turkey; ^3^Division of Food, Nutrition and Public Health, University of Westminster, London, United Kingdom; ^4^Department of Medicine, Surgery and Dentistry “Scuola Medica Salernitana”, University of Salerno, Baronissi, Italy; ^5^College of Medicine, QU Health, Qatar University, Doha, Qatar

**Keywords:** children, bone health, vitamin D, chronic disease, outcomes

The metabolism of vitamin D relies predominantly upon the synthesis of cholecalciferol (vitamin D3) in the epidermis following exposure to sunlight (specifically ultraviolet B rays) and the subsequent hydroxylation to active forms in the liver and kidney (1). Vitamin D can also be sourced from natural dietary sources such as fatty fish or from supplemented foods such as bread or cow's milk.

One of the primary roles of vitamin D is the regulation of intestinal absorption of cations such as calcium and magnesium (1, 2). Inadequate vitamin D results in less intestinal absorption and consequent adverse effects on bone health. Vitamin D deficiency may present with rickets in early childhood or osteomalacia at any age (2).

Besides bone health, there is increasing interest in other biological roles of Vitamin D through interaction with the vitamin D receptor expressed in several tissues (3, 4). These include anti-infective and immunoregulatory roles and have led vitamin D status to be linked with the etiology and severity of various chronic health conditions. This Research Topic aimed to draw together reports focusing on aspects of vitamin D relevant to child health overall and also to specific disease states in childhood.

## Epidemiology of Vitamin D in Infants and Children: Implications for Child Health

Chen et al. undertook a cross-sectional assessment of vitamin D status in more than 1,500 preschoolers in Hangzhou, China. Only 36% of these children were vitamin D sufficient (level ≥ 30 ng/ml), while 11.4% were deficient and 52.6% insufficient. The children with obesity in this group had the lowest mean levels of vitamin D and the highest rate of deficiency. Vitamin D levels were associated with increased risk of dental caries. Furthermore, vitamin D levels were lower in older compared to younger children. This work demonstrated high rates of inadequate vitamin D these children, with status associated with two key childhood outcomes.

Kondratyeva et al. assessed the vitamin D status of 333 children from three Russian regions (Moscow, Krasnoyarsk and Stavropol). Overall, the mean vitamin D level was 32.74 (±17.47) ng/ml: 45.5% of the children had normal levels. The levels were dependent upon the residential location of the children and the respective sunshine hours. Vitamin D status was not associated with polymorphisms in key genes involved with vitamin D synthesis, hydroxylation or transport.

Other publications have examined the association between growth and vitamin D status in children, as noted by Song et al., in their systematic review and meta-analysis. After assessment, the authors included seven studies conducted in four countries (Tanzania, Iran, India and Ecuador). Vitamin D status was associated with wasting but not stunting in the 7,624 children included (summary risk estimate = 1.3; 95% confidence interval 1.04–1.62). This report highlights the importance of vitamin D in children's growth, but did not include longitudinal or interventional studies.

## Effects of Vitamin D Status Upon Calcium Metabolism and Bone Health

Two of the included reports focused on the key role of vitamin D in calcium absorption and bone health.

Mohamed et al. prospectively evaluated calcium and vitamin D status in 26 premature infants, a group particularly at risk of developing metabolic bone disease. Thirteen of the infants developed osteopenia. Daily protein and vitamin D intakes (but not calcium intake) were lower in those who developed osteopenia. Similarly, vitamin D levels at 6 weeks of chronological age were also lower in those with osteopenia. This report emphasized key aspects of bone health in this vulnerable group of infants, and provides a foundation for preventative and interventional studies.

In a retrospective chart review, Gurevich et al., described the features of a different aspect of vitamin D status: these 10 children were characterized by elevated vitamin D. The children were shown to have hypercalcemia or hypercalciuria and suppressed parathyroid hormone levels. Four of the children had mutations in either SLC34A or CYP24A1 genes. With intervention, the children had improved serum vitamin D and calcium levels, but not all had resolution of hypercalciuria or nephrocalcinosis.

## Vitamin D and Specific Disease States

The potential impact of vitamin D upon the pathogenesis or outcome of three different conditions were considered.

Firstly, Li et al. undertook a systematic review and meta-analysis of studies that have evaluated vitamin D status in children diagnosed with a urinary tract infection (UTI). Six studies (339 children with a UTI and 306 healthy controls) were included. Overall, vitamin D status was linked with the risk of developing a UTI.

Vitamin D's immunoregulatory roles may contribute to the development of autoimmune conditions. Zou et al. specifically reviewed the data supporting a role of vitamin D in the development of arthritis. They also reviewed the published studies evaluating the benefits of vitamin D supplementation upon disease outcomes. The authors highlighted particular avenues for future investigation that would further clarify this area of interest.

Ye et al. showed a relationship between baseline vitamin D status and outcomes of cardiac surgery. Nine hundred children with congenital heart disease undergoing cardiac surgery were included. The median vitamin D level preoperatively was 24 ng/ml: a third of the children were vitamin D deficient.

The children with vitamin D deficiency were more likely to have greater inotrope requirements 24 h postoperatively (Odds Ratio 2.27). Although this report highlighted the relevance of vitamin D in this context, it did not examine other outcomes or consider the mechanism of this observation.

## Conclusions

Together the articles comprising this special issue provide insights and updates into various aspects of vitamin D in child health and disease. These reports also serve to highlight the many gaps in current understanding and raise a foundation for future and ongoing endeavors. These reports reinforce the high prevalence of vitamin D deficiency and insufficiency in the general population and the safety of this low-cost molecule and opens new perspectives regarding extra-skeletal effects of vitamin D. We thank all the Authors for their contributions dedicated to an old pro-hormone whose potential interests beyond mineral metabolism are still being investigated.

## Author Contributions

All authors listed have made a substantial, direct, and intellectual contribution to the work and approved it for publication.

## Conflict of Interest

The authors declare that the research was conducted in the absence of any commercial or financial relationships that could be construed as a potential conflict of interest.

## Publisher's Note

All claims expressed in this article are solely those of the authors and do not necessarily represent those of their affiliated organizations, or those of the publisher, the editors and the reviewers. Any product that may be evaluated in this article, or claim that may be made by its manufacturer, is not guaranteed or endorsed by the publisher.
